# A pilot study on the effects of olfactory stimulation with white musk aromatic oil on psychophysiological activity: a crossover study

**DOI:** 10.1038/s41598-024-83887-2

**Published:** 2025-01-11

**Authors:** Fadilla Zennifa, Taisuke Nakashima, Yanli Xu, Saki Koshio, Erika Tomimatsu, Akiko Isa, Katsuya Satake, Fumi Kishida, Kuniyoshi Shimizu

**Affiliations:** 1 Department of Agro‑Environmental Sciences, Faculty of Agriculture, Kyushu University, 744 Motooka, Nishi‑ku, Fukuoka, 8190395 Japan; 2 Nextday Co., Ltd, 29-36 Sakuragaoka-cho, Shibuya-ku, Tokyo, 150-0031 Japan; 3 Department of Medical Laboratory Science, Faculty of Health Sciences, Junshin Gakuen University, 1-1-1 Chikushigaoka, Minami-ku, Fukuoka, 815-8510 Japan

**Keywords:** White musk, Aromatic oil, Electroencephalogram, Psychophysiology, Electrocardiogram (ECG), GC-MS, Attention, Neuroscience, Psychology, Neurophysiology

## Abstract

Studies on the compounds of aromatic oils and their effects on psychophysiological changes in humans are often conducted separately. To obtain better validation, a suitable protocol is needed that can be extrapolated to large-scale olfactory stimulation experiments. Unfortunately, this type of study is still rarely performed. In this situation, we propose a randomized crossover pilot study on olfactory stimulation with aromatic oils in relation to changes in psychophysiological activity by focusing on white musk aromatic oil due to its popularity in the community. Chemical profiling by TDU-GC-MS (thermal desorption gas chromatography/mass spectrometry) was performed to understand the compounds of the aromatic oils presented. To understand the changes in the participants’ impressions and mood states, POMS 2 (Profile of Mood States 2nd Edition) and VAS (Visual analogue scale) were performed in addition to physiological evaluation by using EEG (electroencephalogram), ECG (electrocardiogram) and salivary amylase measurements. The proposed pilot study showed “gorgeous”, “sweet”, and “like” impression toward white musk aromatic oil under VAS evaluation. Mood evaluation under POMS 2 variables such as Fatigue-Inertia (FI), Tension-anxiety (TA) and TMD (total mood disturbance) were significantly decreased under white musk aromatic oil inhalation. Under current protocol, we can also see the changes in autonomic activity and brain activity during olfactory stimulation. This pilot study could be the first step towards a larger sample size experiment on olfactory stimulation. This experiment has been registered to UMIN Clinical Trials Registry with register ID : UMIN000051972 on 24/08/2023.

**Supplementary Information:**

The online version contains supplementary material available at 10.1038/s41598-024-83887-2.

## Introduction

In recent years, aromatic oil has attracted attention from various fields such as environmental and cosmetics. Aromatic oils are produced from various sources, including animal, plant, and synthetic fragrances. Research on the aromatic oil’s compounds and their effects on the human body are often conducted separately. Okada et al.^1^ mentioned the effect of aromatic oil toward autonomic nervous system during exercise. Their study showed that compared with aromatic oil, the heart rate of the participants showed a later recovery. At the same time, it would be better if the olfactory stimuli also contained information about the chemical compounds of the aroma to be used. Since the analysis of chemical and psychophysiological effects requires multidisciplinary skills, the combination of the two studies is rarely done. Therefore, our proposed study became a pilot study to explore the effect of aromatic oil on olfactory stimulation. We conducted a randomized crossover pilot study to determine how best to conduct a large-scale research project in an exploratory study of olfactory stimulation with aromatic oils and olfactory stimulation at rest with closed eyes. To understand what compounds are contained in the aromatic oil to be used in this olfactory stimulation, this study used TDU-GC-MS (thermal desorption device combined with gas chromatography and mass spectrometry). Thermal desorption is a technique that uses heat to increase the volatility of contaminants and separated them from a solid matrix without combusting the medium or contaminants^2^. This is the background why we used the technique to find the compound of aromatic oil and do the chemical profiling. Chemical profiling is needed to gain the understanding of what compound are contained in the aroma and to investigate the effect of aroma on human psychophysiological activity.

The aroma’s journey into the human body is first to the brain regions that process emotion and memory. Therefore, measuring brain activity becomes crucial to see the effect of white musk fragrance on human psychophysiology. Electroencephalography (EEG) becomes a suitable measurement device since it is a non-invasive method and has no side effects in the use of this electrical brain activity monitoring to the participants. The data of EEG its selves can be extracted into various features, from linear to nonlinear features^3^. The classification of EEG features based on its frequency could be referred to International Federation of Clinical Neurophysiology (IFCN) 2017^4^. Where the theta (ø)band is in the range of 4 to less than 8 Hz, alpha band is in the range 8 to 13 Hz, beta band is in the range 14 to 30 Hz and gamma band is distinguish in the frequency more than 30 Hz to 80 Hz. Gamma band in this study is divided into two categories, low gamma (31–59 Hz) and high gamma (65–80 Hz). The frequency band features of EEG data and its dominant power activities electrode’s location often correlated to the mental states of the human. We also explored the nonlinear features of neural activity which is called the slope of 1/f fluctuations. Several studies found there are information hidden under the slope of 1/f instead just seeing it as a noise without neuronal information. Gao et al.^5^ and Waschke et al.^6^ mentioned that excitation (E) and inhibition (I) of neural circuit can be estimated from the slope of 1/f. Where Donoghue^7^ et al. providing the algorithm for 1/f analysis that could be used for aging related analysis. Voytek et al. in 2015^8^, explored the correlation between the slope of 1/f with the age, founding of their study mentioned that aging is associated with a flatter 1/f. However, until now, there is no other research that investigates the effect of fragrance on the slope of 1/f. This represents our novelty point, as it will be the first pilot study to investigate the slope of 1/f change for aromatic oil inhalation.

To measure the effect of aromatic oil on human physiology, this study also monitored the autonomic activity of the heart. Electrocardiography (ECG) is used to monitor the electrical activity of the heart through the cycles of the heart using an electrode placed on the skin. The features from the ECG data consisted of the sympathetic nervous system (SNS) and parasympathetic nervous system (PNS), which are reflected in the low frequency (LF) and high frequency (HF) bands in heart rate variability (HRV), while the sympathovagal balance is shown on the ratio of the powers in these frequency bands, the so-called LF-HF ratio (LF/HF)^9^. Another sympathetic activity measurement that has correlation with the stress is salivary amylase. The significant differences were found between the stress and the rest condition in salivary alpha-amylase in Nater et al.^10^ research that created the change of alpha amylase as stress index biomarker^11–13^.

Visual analog scale (VAS) was used to measure the participants’ impression of the presented aroma. The mood changes of the participants are observed by using Profile of Mood States (POMS) questionnaire. VAS is a visual-based scale that typically measures participant impressions of stimuli and participant feelings based on past references^14^. At the same time, The Profile of Mood States Second Edition (POMS2) Japanese version is used to evaluate the mood states of the participant that also been used by several studies such as evaluation of mood^15,16^ .

Due to the many types and characteristics of aromatic oils, in this study we focused on exploring the effects of white musk aromatic oil. White musk aromatic oil was chosen as the experimental material because this aromatic oil is popular among the public. From the various evaluation literature, ours is the first pilot study to investigate the effects of white musk aromatic oils, including investigating the effects of chemical compounds contained in aromatic oils on human psychophysiology. We are also the first team to investigate the changes in 1/f slope in response to fragrance.

## Results

### Chemical profiling in fragrance olfactory stimulus

This study used white musk aromatic oil as an olfactory stimulus. In the qualitative analysis, several peaks were detected in the white musk aromatic oil. This study used diethylene glycol monoethyl ether (15.7%) and isopropyl myristate (4.7%) as solvents. The component with the largest peak area was linalool (11.9%; detected peak area in the total peak area), and the others were hedione (8.3%), galaxolide(5.2%), α-hexylcinnamaldehyde (4.6%), benzyl acetate (3.7%), and tonalide (1.6%). Galaxolide and tonalide, were selected for quantitative analysis because these components are the main characteristic of white musk aromatic oil. The concentrations of galaxolide and tonalide were 34.8 ± 1.9 mg/mL and 15.0 ± 0.8 mg/mL, respectively.

### Psychological evaluation (survey)

#### Participant impression toward presented aromatic oil

The VAS was used to quantify the fragrance impression of a room using a total of 21 items, including adjectives and verbs expressing fragrance characteristics (Table 1). In the before-and-after comparison of the white musk aromatic oil group, the impression of “sour” was significantly reduced after compared to before (*z* = -2.251, *p* = 0.028). On the other hand, no significant difference was found in the before-after comparison of “sour” in the unscented group and in the comparison of the amount of change between the unscented group and the white musk aromatic oil group.

**Table 1:** Statistic value of 21 VAS variables.

No	Variable	Value	Unscented			White Musk			(Post-pre)		
			Pre	Post	p	Pre	Post	p	Unscented	White Musk	p
1	Like	Average	41.2	43.5	0.473	48.9	67.4	0.003	2.3	18.5	0.01
		SD	19.107	22.697		22.098	23.543		9.719	14.37	
2	Bitter	Average	14.4	9.7	0.05	9.7	9.9	0.93	-4.7	0.2	0.293
		SD	22.853	17.263		23.828	19.296		9.9	7.036	
3	Sweet	Average	14.4	12.7	0.651	18.4	53.5	0.011	-1.7	35.1	0.026
		SD	20.023	22.131		28.826	27.448		11.48	34.857	
4	Weak	Average	43.2	46	0.678	42.4	26	0.181	2.8	-16.4	0.176
		SD	39.793	40.792		40.536	26.953		20.666	35.756	
5	Astringent	Average	19	9.2	0.268	10	5.6	0.26	-8.5	-4.4	0.625
		SD	28.548	9.126		12.737	7.412		22.658	11.578	
6	Sour	Average	6	7.4	0.498	1.9	8.1	0.028	1.4	6.2	0.29
		SD	12.374	9.442		1.853	11.542		6.275	10.304	
7	Strong	Average	7	8.6	0.551	14.3	25.5	0.077	1.6	11.2	0.157
		SD	12	15.586		21.644	21.706		4.3	17.706	
8	Roasty	Average	12.3	9	0.307	8.2	13.2	0.411	-3.3	5	0.191
		SD	15.564	9.877		10.031	18.293		9.627	18.348	
9	Fresh	Average	29.6	32.2	0.642	29.4	37.2	0.337	2.6	7.8	0.443
		SD	29.836	25.446		32.911	22.503		17.096	24.33	
10	Relaxing	Average	56.7	51.2	0.545	60.5	57.3	0.374	-5.5	-3.2	0.832
		SD	28.918	29.257		24.681	27.125		27.629	10.809	
11	Clear	Average	58.7	48.8	0.182	58.3	49.6	0.192	-9.9	-8.7	0.901
		SD	28.123	26.347		28.91	28.972		21.656	19.522	
12	Comfortable	Average	49.4	50.5	0.575	61.4	60	0.716	1.1	-1.4	0.717
		SD	21.711	29.209		21.864	25.491		18.764	11.796	
13	Breezy	Average	30.8	43.2	0.097	41.6	53.7	0.22	12.4	12.1	0.976
		SD	24.138	30.521		27.794	29.307		21.136	29.019	
14	Refreshing	Average	35.4	41.8	0.461	44	46.5	0.805	6.4	2.5	0.699
		SD	32.452	27.345		32.152	31.26		26.311	31.167	
15	Elegant	Average	24.2	25.9	0.691	28.9	48.4	0.057	1.7	19.5	0.075
		SD	26.931	26.735		32.088	30.126		13.073	28.313	
16	Modern	Average	20.7	15.8	0.358	31.8	46.8	0.299	-4.9	14	0.169
		SD	17.664	24.147		26.288	29.287		16.003	40.169	
17	Smelly	Average	2.9	3.3	0.309	4.8	3	0.443	0.4	-1.8	0.316
		SD	3.929	4.322		5.77	3.944		1.174	7.099	
18	Nostalgic	Average	13.6	11.5	0.905	21.6	15.1	0.385	-2.1	-6.5	0.643
		SD	20.195	11.148		19.755	15.843		12.793	22.496	
19	Mild	Average	30.6	42.6	0.028	38.2	52	0.212	12	13.8	0.856
		SD	24.604	29.083		28.221	27.297		14.461	32.519	
20	Calming	Average	52.2	51	0.8	54.2	61.8	0.262	-1.2	2.6	0.275
		SD	25.879	28.123		32.341	27.684		14.528	20.084	
21	Gorgeous	Average	7.7	15.9	0.295	14.7	49.8	0.009	8.2	35.1	0.04
		SD	10.231	21.738		25.224	29.78		16.565	33.805	

In a before and after comparison of the white musk group, the impressions of the three items “like”, “sweet”, and “gorgeous” increased significantly after compared to before (*t*(9) = -4.071, *p* = 0.003, *t*(9) = -3.184, *p* = 0.011, *t*(9) = -3.283, *p* = 0.009). On the other hand, no significant differences were found in the before and after comparisons of the unscented group on these three items. In addition, when comparing the unscented group and the aromatic oil group in terms of the amount of change in each item, the amount of change in the aromatic oil group was significantly greater than the amount of change in the unscented group for the three items “like”, “sweet”, and “gorgeous”. (*t* (9) = -3.232, *p* = 0.010, *t*(9) = -2.657, *p* = 0.026, *t*(9) = -2.397, *p* = 0.040). These results showed that the white musk that be used as aromatic oil has strong impressions of “like”’, “sweet” and “gorgeous”.

#### Mood changes in response to aromatic oil

Seven mood scales and the associated Total Mood Disturbance (TMD) score were quantified using the short version of the POMS2 (Table 2). A pre-test comparison within each condition revealed that the Confusion-Embarrassment (CB) and Fatigue-Inertia (FI) scales were measured in both the unscented and aromatic oil groups, and post-test scores were compared with pre-test scores. showed a significant decrease (CB: *t*(9) = 2.290, *p* = 0.048; *t*(9) = 2.363, *p* = 0.042, FI: *z* = 2.201, *p* = 0.036; *t*(9) = 4.348, *p* = 0.002). The ``Tension-Axiety (TA)’’ and ``Total Mood Disturbance (TMD)’’ scales showed a significant decrease in post-test scores compared to pre-test scores in the white musk (aromatic oil) group (TA: *t* (9) = 2.941, *p* = 0.016, TMD: *t*(9) = 2.580, *p* = 0.030). There was no significant difference between the unscented group and the white musk aromatic oil group in terms of the amount of change in each item.

**Table 2:** Statistic value of POMS 2.

No	Variable	Value	Unscented			White Musk			(Post-pre)		
			Pre (average)	Post (average)	p	Pre (average)	Post (average)	p	Unscented (average)	White Musk (average)	p
1	AH	Average	39.6	39.5	0.943	40	38.2	0.053	-0.1	-1.8	0.343
		SD	1.578	3.567		2.309	0.632		4.332	2.394	
2	CB	Average	47.4	42.1	0.048	44.7	41.3	0.042	-5.3	-3.4	0.458
		SD	6.168	3.542		6.093	4.138		7.319	4.551	
3	DD	Average	42.3	41.8	0.485	42.1	41.2	0.371	-0.5	-0.9	0.565
		SD	1.767	1.398		2.234	0.632		2.173	2.234	
4	FI	Average	46.4	40.4	0.036	46.9	39.1	0.002	-6	-7.8	0.435
		SD	7.849	6.004		5.238	3.872		7.557	5.673	
5	TA	Average	44.1	40.4	0.271	42.1	39.3	0.016	-3.7	-2.8	0.8
		SD	11.08	5.985		5.322	4.398		10.209	3.011	
6	VA	Average	61.5	56.3	0.055	61.3	56.3	0.076	-5.2	-5	0.959
		SD	8.81	12.139		7.587	9.417		7.465	7.874	
7	F	Average	55.9	55.4	0.717	58.5	56.7	0.118	-0.5	-2.1	0.519
		SD	10.322	11.843		13.431	13.409		4.223	3.843	
8	TMD	Average	41	38.9	0.106	40.3	37.8	0.03	-2.1	-2.5	0.702
		SD	4.967	3.348		3.622	3.011		3.695	3.064	

### Physiological evaluation

#### Salivary amylases result in response to aromatic oil inhalation

**Fig. 1:**
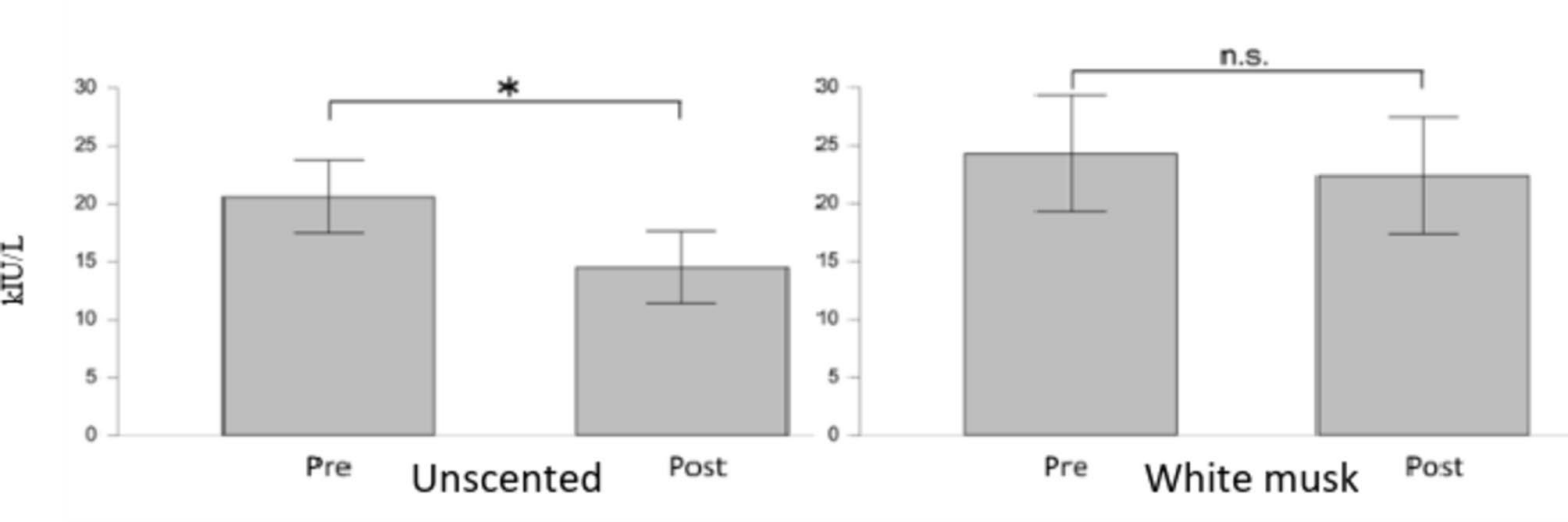
Alpha amylase activity in unscented and White Musk. In this experiment, we confirmed that there was no significant change under aromatic oil inhalation, but on the other hand, there were significant changes in salivary alpha-amylase when the participant under the unscented condition.

A before and after comparison of the saliva test results showed a significant difference in the post-measurement of the unscented group *t* (9) = 2.262, *p* = 0.012 as shown in Fig. 1. No significant difference was observed in the White Musk group *t*(9) = 2.262, *p* = 0.56.

#### Autonomic activities change after the aromatic oil inhalation

Since ECG has a slower response compared to brain activity, we found that after 10 min of white musk aromatic oil presentation, LF/HF in the white musk (white musk aromatic oil) group was significantly decreased compared to the unscented group (*t* (8) = 3.518, *p* = 0.024), as shown in Table 3.

**Table 3:** Electrocardiogram result during experiment is conducted.

Condition	Variables	Unscented (mean ± SD)	White musk (mean ± SD)	t	df	p*
Before olfactory stimulation presented	RRI	839.756 ± 122.973	849.4 ± 122.973	-0.535	8	1.
	LF	32.811 ± 11.729	30.978 ± 12.715	0.576	8	1.
	HF	18.278 ± 4.176	18.911 ± 6.048	-0.427	8	1.
	LF/HF	1.978 ± 0.816	1.727 ± 0.546	1.386	8	0.609
Olfactory stimulation is attached on the wall	RRI	876.444 ± 122.143	887.089 ± 118.367	-0.483	8	1.
	LF	43.456 ± 26.047	33.589 ± 17.539	2.447	8	0.12
	HF	22.4 ± 8.397	20.178 ± 7.693	0.891	8	1.
	LF/HF	2.081 ± 0.774	1.9 ± 0.83	1.943	8	0.264
After 10 min inhalation and stimulus ejected from the wall	RRI	868.8 ± 122.693	872.678 ± 125.108	-0.157	8	1.
	LF	46.244 ± 26.294	37.2 ± 18.57	1.473	8	0.537
	HF	21.178 ± 6.999	20.078 ± 7.376	0.413	8	1.
	LF/HF	2.3 ± 0.884	2.023 ± 0.807	3.518	8	0.024

*p Bonferroni correction.

#### Brain analysis result

##### Power spectrum frequency band (PSD)

**Fig. 2:**
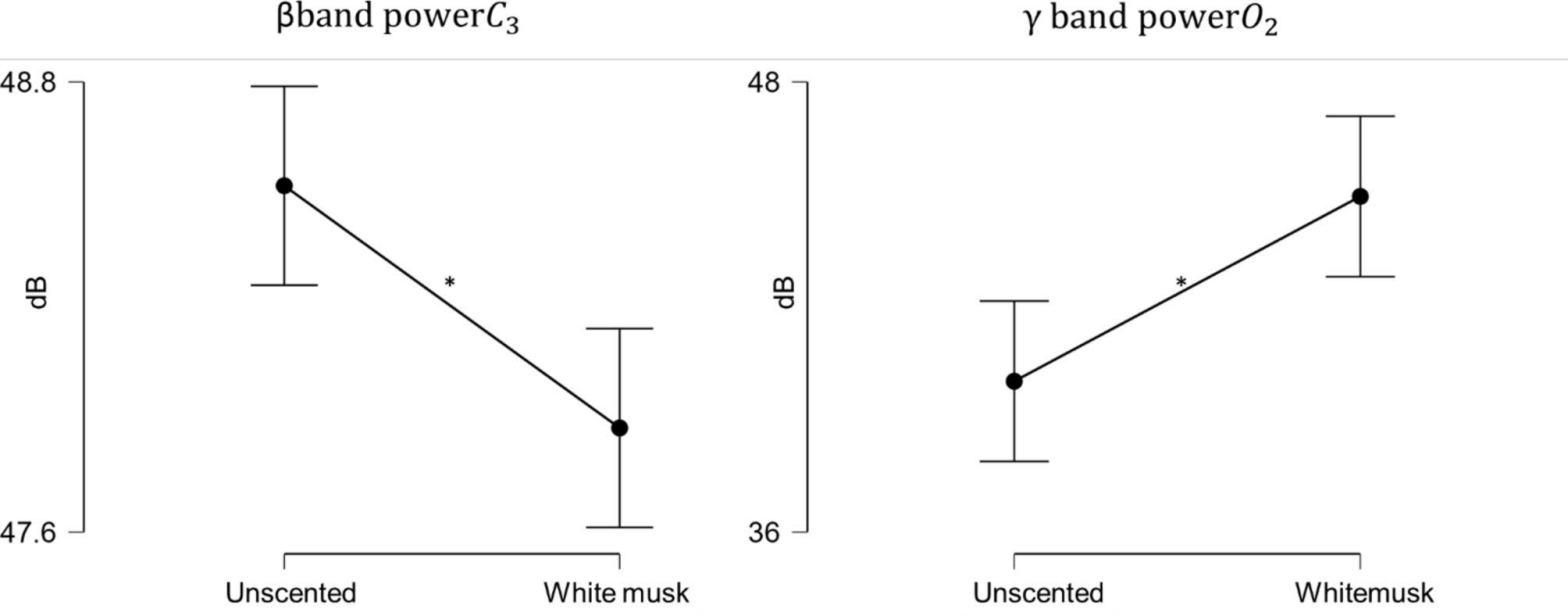
The changes of beta and gamma band in specific brain location. The left picture shows the changes on the beta band on the left central area (C3) between unscented and white musk for 10 min inhalation and the right picture shows the increase on the gamma band on the right hemisphere of the occipital between unscented and white musk for 10 min inhalation.

**Fig. 3:**
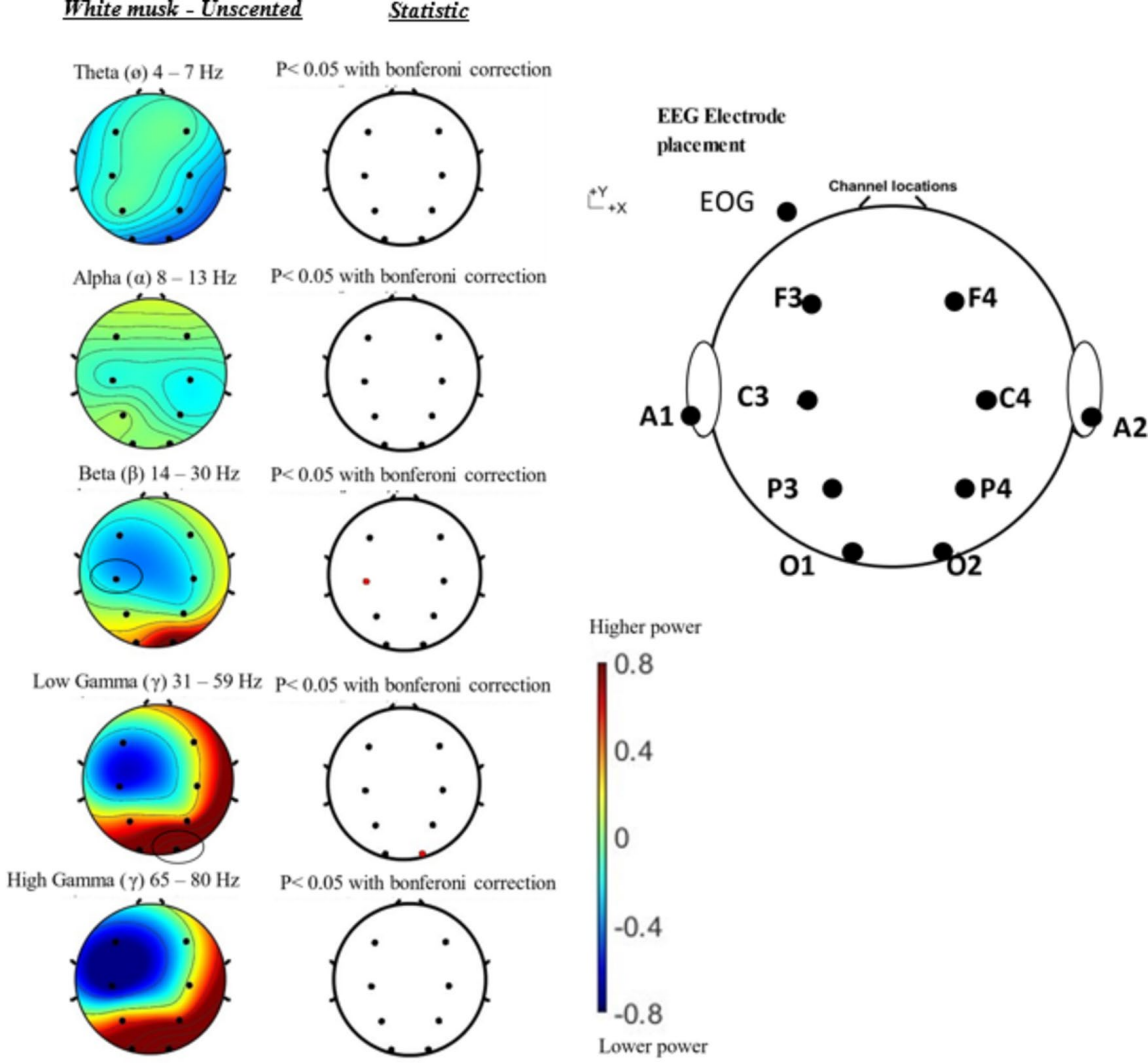
Brain topography from 8 channels between white musk- unscented. Subtraction between white musk-unscented PSD from 8 channel EEG, the red dot on the statistic (left image) means where the value is significant, red-ish color in the brain topography showed higher power, where blue-ish showed lower power under aromatic oil inhalation (right).

We performed EEG power spectrum frequency analysis and mapped the potential distribution of specific EEG activity across the scalp from 8 representative channels. As a result of visual examination of areas with specific changes between groups in each brain wave frequency band after the Bonferroni correction, changes were observed in the beta band of the left central region (C3 region) (*t* (8) = 3.972, *p* = 0.032) and the gamma band of the right occipital region (O2 region) ((*t* (8) = -3.757, *p* = 0.048) as shown in Fig. 2. This study also presented the brain topography mapping of 8 representative channels of the brain area in Fig. 3. The brain topography mapping based on the subtraction of each individual average power spectrum for easier interpretation since the electrode used are 8 channels. The color indicates the brain location that has dominant activity with red-ish color showed the higher power activities in white musk aromatic oil where the blue-ish color showed a lower power activity under white musk aromatic oil inhalation. Where the statistical part shows the paired t-test comparison between power spectrum of white musk inhalation and unscented with Bonferroni comparison to each electrode location. All electrode placement measurement values and their changes to experimental conditions can be seen in Table 4.

**Table 4:** The changes of brain activity during experiment.

	Before olfactory stimulation presented			Olfactory stimulation is attached on the wall				After 10 min inhalation and stimulus ejected from the wall	
	Unscented (mean ± SD)	White Musk (mean ± SD)	p*	Unscented (mean ± SD)	White Musk (mean ± SD)	p*	Unscented (mean ± SD)	White Musk (mean ± SD)	p*
Theta (θ)									
F3	56.907 ± 2.22	56.875 ± 1.59	1	57.165 ± 2.055	56.91 ± 1.646	1	57.255 ± 1.916	56.996 ± 1.656	1
F4	57.305 ± 2.393	57.413 ± 1.665	1	57.61 ± 2.185	57.491 ± 1.545	1	57.732 ± 2.166	57.689 ± 1.594	1
C3	57.071 ± 2.05	56.883 ± 1.566	1	57.263 ± 1.788	57.011 ± 1.508	1	57.415 ± 1.767	57.062 ± 1.529	1
C4	57.239 ± 2.2	56.789 ± 1.905	1	57.532 ± 2.052	57.028 ± 1.769	1	57.742 ± 2.148	57.139 ± 1.711	1
P3	56.932 ± 2.333	56.896 ± 1.919	1	57.002 ± 2.002	56.877 ± 1.636	1	57.228 ± 2.016	56.82 ± 1.544	1
P4	57.015 ± 2.586	56.356 ± 1.951	0.984	57.18 ± 2.244	56.615 ± 1.788	1	57.4 ± 2.306	56.528 ± 1.652	1
O1	56.186 ± 2.854	55.774 ± 2.079	1	56.614 ± 2.557	55.882 ± 1.812	1	57.018 ± 2.725	56.235 ± 1.885	1
O2	56.478 ± 2.845	55.738 ± 2.605	0.672	56.755 ± 2.52	55.789 ± 2.037	0.808	57.104 ± 2.631	56.164 ± 2.016	1
Alpha (α)									
F3	57.664 ± 2.804	57.674 ± 2.346	1	56.544 ± 3.328	56.537 ± 2.686	1	56.423 ± 3.471	56.323 ± 2.898	1
F4	58.144 ± 2.925	58.212 ± 2.388	1	57.17 ± 3.337	57.174 ± 2.671	1	57.082 ± 3.426	56.987 ± 2.85	1
C3	58.856 ± 2.98	58.728 ± 2.457	1	57.803 ± 3.34	57.504 ± 2.786	1	57.651 ± 3.56	57.347 ± 2.888	1
C4	59.241 ± 2.775	58.828 ± 2.366	1	58.26 ± 3.246	57.853 ± 2.806	1	58.094 ± 3.425	57.693 ± 2.868	1
P3	60.34 ± 3.568	60.475 ± 2.722	1	58.912 ± 3.627	59.018 ± 2.997	1	58.514 ± 3.921	58.834 ± 3.064	1
P4	60.202 ± 3.155	60.174 ± 1.834	1	59.068 ± 3.563	58.748 ± 2.628	1	58.669 ± 3.763	58.45 ± 2.709	1
O1	61.574 ± 3.496	61.307 ± 2.483	1	59.786 ± 3.515	59.798 ± 2.58	1	59.547 ± 3.543	59.433 ± 2.52	1
O2	61.872 ± 3.477	61.752 ± 2.629	1	60.169 ± 3.655	60.225 ± 2.995	1	59.908 ± 3.725	59.853 ± 2.782	1
Beta (β)									
F3	48.795 ± 2.564	48.017 ± 2.351	1	48.113 ± 2.402	47.466 ± 2.333	0.32	48.877 ± 2.787	47.605 ± 2.36	1
F4	49.611 ± 2.821	49.13 ± 3.406	1	48.888 ± 2.531	48.784 ± 3.28	1	49.506 ± 2.561	48.836 ± 3.282	1
C3	49.21 ± 2.548	48.529 ± 2.131	1	48.523 ± 1.808	47.878 ± 2.196	0.032	49.181 ± 2.712	47.923 ± 2.227	1
C4	50.134 ± 2.954	49.265 ± 2.473	1	48.94 ± 2.161	48.561 ± 2.491	1	49.481 ± 2.842	48.575 ± 2.738	1
P3	49.499 ± 2.607	49.545 ± 2.183	1	48.543 ± 2.316	48.717 ± 2.257	1	48.842 ± 2.505	48.756 ± 2.322	1
P4	49.912 ± 2.671	49.427 ± 1.912	1	48.775 ± 2.476	48.703 ± 1.92	1	49.222 ± 2.811	48.731 ± 2.111	1
O1	50.534 ± 2.403	51.033 ± 2.816	1	50.117 ± 1.91	50.935 ± 2.88	1	50.396 ± 1.878	51.281 ± 2.75	1
O2	50.585 ± 2.38	51.573 ± 2.714	1	49.933 ± 2.273	51.96 ± 2.681	0.384	50.277 ± 2.208	52.304 ± 2.987	1
Low gamma (γ)									
F3	39.156 ± 4.277	37.703 ± 2.789	1	37.459 ± 3.151	36.543 ± 2.414	1	39.097 ± 4.993	36.961 ± 2.72	1
F4	39.997 ± 4.178	39.733 ± 5.669	1	38.15 ± 3.32	38.605 ± 5.639	1	39.249 ± 4.144	38.529 ± 5.651	1
C3	38.937 ± 3.508	37.539 ± 2.135	1	37.808 ± 2.315	36.647 ± 1.974	1	39.361 ± 4.66	36.953 ± 2.443	1
C4	40.416 ± 4.944	39.67 ± 3.948	1	38.192 ± 2.226	38.635 ± 3.699	1	38.981 ± 4.849	38.494 ± 4.218	1
P3	38.466 ± 2.777	38.397 ± 2.43	1	37.379 ± 1.911	38.325 ± 2.298	1	38.622 ± 2.8	38.683 ± 2.392	1
P4	38.913 ± 3.586	38.528 ± 1.903	1	37.281 ± 1.958	38.276 ± 1.689	1	38.516 ± 3.658	38.625 ± 2.156	1
O1	40.487 ± 3.608	41.994 ± 4.652	1	40.915 ± 2.953	43.081 ± 4.401	1	41.957 ± 2.953	44.012 ± 4.075	1
O2	39.599 ± 3.234	43.765 ± 3.081	0.216	40.022 ± 2.451	44.947 ± 3.06	0.048	41.283 ± 1.948	45.473 ± 3.69	1
High gamma (γ)									
F3	37.298 ± 5.756	35.428 ± 3.53	1	35.19 ± 4.692	33.597 ± 2.881	0.944	36.874 ± 7.049	33.98 ± 3.549	1
F4	38.109 ± 5.653	37.161 ± 6.254	1	35.659 ± 4.389	35.501 ± 6.18	1	36.447 ± 5.467	35.258 ± 6.243	1
C3	36.599 ± 5.491	34.893 ± 2.849	1	35.338 ± 3.746	33.581 ± 2.41	1	37.093 ± 6.768	33.936 ± 3.239	1
C4	37.995 ± 6.404	37.062 ± 4.959	1	35.469 ± 3.405	35.815 ± 4.385	1	36.188 ± 6.111	35.558 ± 4.999	1
P3	35.981 ± 3.841	35.767 ± 2.385	1	34.58 ± 2.36	35.623 ± 2.463	1	36.266 ± 4.502	36.044 ± 2.818	1
P4	35.949 ± 4.916	36.227 ± 2.122	1	34.315 ± 2.615	35.681 ± 1.296	1	35.746 ± 4.875	36.11 ± 1.981	1
O1	38.18 ± 4.092	40.075 ± 4.766	1	38.546 ± 3.554	41.122 ± 4.471	1	39.809 ± 3.919	42.081 ± 4.176	1
O2	36.94 ± 4.281	42.213 ± 2.983	0.208	37.792 ± 2.764	43.066 ± 2.846	0.056	39.316 ± 2.459	43.514 ± 3.378	0.144

*p Bonferroni correction.

##### 1/f fluctuations result

**Fig. 4:**
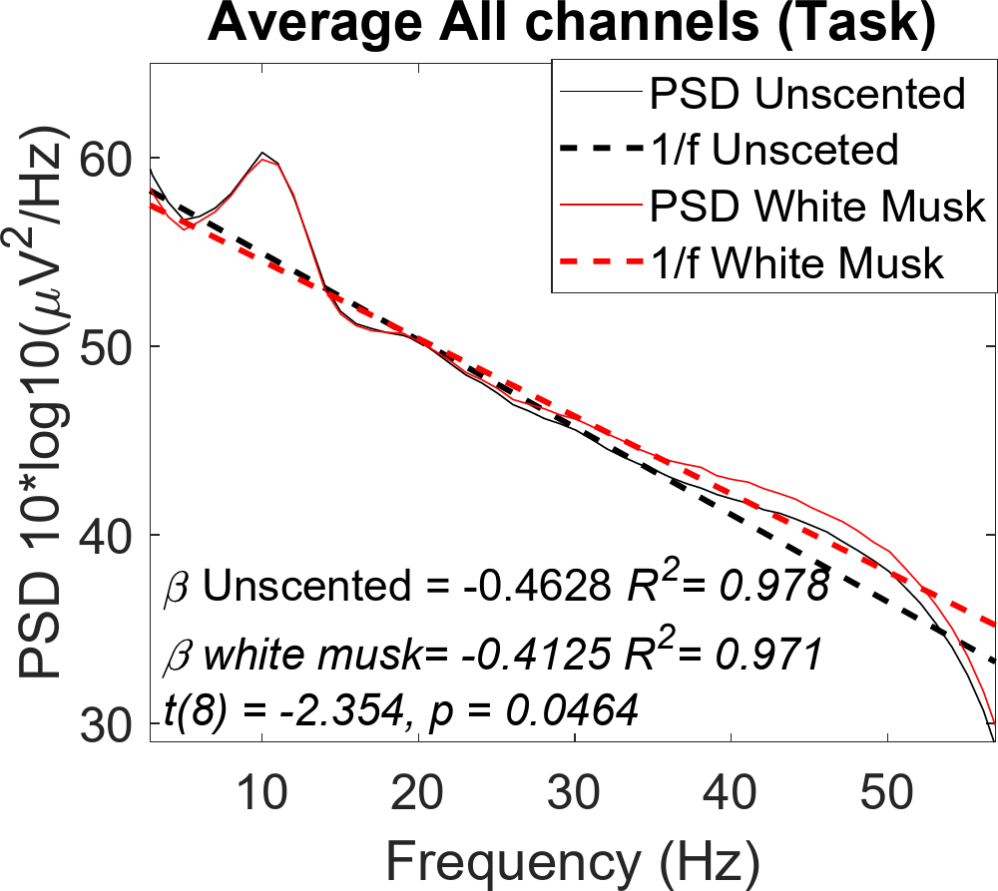
1/f slope and PSD during inhalation of aromatic oil within 10 min inhalation. 1/f activity was shown to be gentler when participants exposed with the aromatic oil comparing with the unscented condition. This phenomenon may occur under the influence of dopamine. When humans like something, they release the hormone dopamine, which causes an increase in vibrational activity in the brain. The larger the oscillation, the greater the force, and the slope tends to be gentler.

1/f is a fitting line (dotted line) of the power spectral density (PSD) versus frequency, and Fig. 4 shows the average PSD results and 1/f from all electrodes of the nine participants analysed. During olfactory stimulus, the slope of 1/f in the White Musk (white musk aromatic oil) group was significantly gentler than that in the unscented group starting from 37 Hz oscillation (*t*(8) = -2.354, *p* = 0.046).

## Discussion

This pilot study experiment used white musk as an olfactory stimulus. The choice of using this fragrance among other fragrances such as lavender and fir that we used to confirm the olfactory function of the participants because white musk is one of the best-selling products in the fragrance category. To get the right amount of white musk in a 4.2 m^3^ room, researchers in this team was conducted several aroma tests when the amount of white musk was in 5 µL, 10 µL, 20 µL, 100 µL before the experiment is conducted to participants. This study found that the fragrance of 10 µL below was appropriate to avoid strong aroma within that specific room size. In the component analysis of white musk, we found that the white musk used in the test contained galaxolide (HHCB) and tonalide (AHTN). In general, Galaxolide gives a pleasant aroma impression based on other research^17^, which is also showed in our current result based on participant answer on VAS survey. While Tonalide is a compound also often used in other fragrances^18^. Knowing the chemical profile of white musk aromatic oils is expected to expand the hypothesis about the possibility of other similar white musk aromatic oils on changes in psychophysiological activity. Some common product white musk aromatic oils can have different chemical profiles, therefore mentioning the compounds contained in the white musk aromatic oil is necessary for clear validation conditions. Other studies sometimes only mention the name of the white musk aromatic oil, but how much white musk aromatic oil is presented is often not mentioned. Through this preliminary study, we hope to be a guide for larger experiments in the future.

Salivary amylase activity is known to have a high correlation with plasma norepinephrine concentration, reflecting sympathetic nerve activity and usually reflecting both mental and physical stress^10,13^. Result in our study showed that under unscented condition, there is significant difference in alpha amylase. But there is no significant changes when the participant inhaling the white musk aromatic oil. This might be correlated with the change of mood of participant under white musk aromatic oil condition. As mentioned in the results, under the condition of white musk aromatic oil, FI (Fatigue-Inertia), TA (Tension-Anxiety), TMD (Total mood disturbance) have decreased significantly. The decreasing of alpha amylase in unscented could be correlated to post awakening effect. The salivary alpha amylase was taken prior the electrode placement and after the olfactory experiment finished. During the experiment, participants closed their eyes for 16 min. Because there was no stimulus from the fragrance that induced aroma awareness, participants could feel sleepier compared to when there was a white musk aromatic oil present in the room. Since the salivary alpha amylase is taking within 10–15 min windows after olfactory experiment finish, this could be the reason why in unscented condition the alpha amylase is significant decrease. Some studies mentioned the decrease of saliva alpha amylase within 10–60 min post awakening and increase or flattened after that window^19–21^. The decrement was not corresponded to the relaxing effect itself is confirm by the participant self-assessment via VAS (Visual analogue scale), where the relaxation variables was not significant difference (*p =* 0.545) in unscented condition. At the same time, Electroencephalogram (EEG) measurements reflect neural oscillations in the central nervous system (CNS) and are directly related to various higher-order cognitive processes, including emotion. Emotion recognition based on brain waves shows great potential because it is difficult to intentionally hide, or unscented internal neural oscillations compared to approaches based on facial expressions or speech. Studies that more comprehensively and systematically explored a wide range of EEG features have shown that data in high frequency bands are more influenced by emotion recognition compared to lower frequency bands. Soleymani et al.^22^. performed a cross-subject emotion recognition task using EEG and gaze data and extracted the power spectral density (PSD) of the EEG and found that the most discriminative feature regarding fluctuations in arousal level was found at the occipital electrodes. Emotional features were found in the beta and gamma bands of temporal electrodes. In this study, fluctuations were observed in the beta waves from central electrodes and the low gamma waves from occipital electrodes. This study also found there is increasing value in positive impressions such as “gorgeous”, “sweet” and “like” white musk aromatic oil toward white musk in the VAS survey. Which this finding is consistent with the report that positive emotional recognition affects low gamma band in right hemisphere^23^. Enhancing the previous sentence, beta band activity during white musk aromatic oil olfactory stimulus showed significantly lower activity in the left central region (area C3) compared to unscented. The lower power in the power spectrum of the β band in left hemisphere area during white musk inhalation comparing with unscented observed in this study probably were a response to the participants’ recognition of the white musk scent during fragrance presentation which has pleasant aroma. Regarding the results of the LF/HF index obtained by frequency analysis of heart rate fluctuations, LF/HF was significantly lower than unscented. This is consistent with the fact that within 10 min white musk presentation, beta-band activity in left hemisphere (C3) area was lower compared to unscented and gamma band is higher in right hemisphere (O2). It was suggested that the scent of white musk that is consisted of galaxolide (HHCB) and tonalide (AHTN) may influence the balance of the autonomic nervous system and give a pleasant feeling^24^ and as indication of wellbeing^25^.

Regarding the fluctuations in γ waves observed when presented with fragrances, studies related to various types of meditation have reported that meditation activates of gamma band (> 30 Hz) mainly in the parietal and occipital regions. Lutz et al.^26^ found increased activity in the low gamma band (25–42 Hz) in the lateral frontal lobe and occipital region during non-directed meditation. Cahn *et al.*^27^. reported that during Vipassana meditation, one of India’s oldest meditation methods, the power value of the low gamma band (35–45 Hz) in the parietal and occipital regions increased significantly. Berkovic et al.^28^ reported an increase in the power value of the low gamma wave band (25–45 Hz) in the occipital region during mindfulness meditation, which has recently attracted attention in the medical and business communities. In this experiment, the white musk fragrance showed higher low gamma wave band activity in the right occipital region (O_2_ region) compared to unscented. This suggests that the patient may have been induced into a deep state of mental activity. At the same time, by high gamma band (65–80 Hz) did not show significant difference when using statistical comparison with Bonferroni correction between electrode placement strengthen the statement that the activity gamma band is not only induced by the aroma, but because the aroma is like (likeable) by the participants. Since the experiment in this study has been done within 16 min with closed eyes, the changes of low gamma band also indicated correlation with the meditation states as mentioned Lutz et al.^26^ that long term meditator high amplitude of gamma band (25–42 Hz), which we specified it as low gamma band in this article. The higher amplitude of gamma band in occipital area is indicate with meditation is also explored by Cahn et al.^27^ and Ferrareli et al.^29^.

Until now, the 1/f power spectrum of brain waves has been regarded as background noise, but the possibility that it contains useful information is being investigated. Dave et al.^30^. state that this 1/f neural noise may reflect the distribution of power spectral density over a wide frequency range, and that it is useful in discussing neuronal activation and aging. Erik J Peterson et al.^31^. state that in diagnosing schizophrenia and predicting the severity of symptoms, the steepness of 1/f can better predict the condition than conventionally focusing on vibration power in each frequency band. In this study, we conducted an evaluation of the effects of white musk scent on generally healthy and young participants, so we cannot address the aging and health issues mentioned above, but we do discuss physiological effects from a wide frequency range. It is possible. In a previous study on the 1/f slope, *Gao et al.*^5^. also noted that the 1/f slope became negative under anaesthesia compared to the awake state. Freeman et al.^32^. showed that the slope of PSD during sleep is steeper than during wakefulness. The present pilot study differs from the previously mentioned article in that instead of comparing unconscious and conscious states (sleep and wakefulness), this research protocol is the first to explore non-periodic brain activity in the wakeful state between different scent conditions. The result showed that the slope of 1/f was significantly gentler when white musk aromatic oil was presented compared with unscented condition. It has been reported that when humans feel that they “like” an object, brain wave activity is activated, the brain wave period becomes shorter, the power spectrum in the high frequency region becomes larger, and the slope of 1/f becomes gentler. In our current assumption, this phenomenon may occur under the influence of dopamine. Based on literature, hormone dopamine related with liking or rewards^33,34^. At the same time, the activation of dopamine causes an increase brain oscillation activity in the brain^35^. The larger the oscillation, the greater the force, and the slope tends to be flatter. Indeed, further study need to be done to explore more the effect of olfactory stimulus toward aromatic oil since this study was applied to small number of participants and limited to white musk aromatic oil. This pilot study is expected can be the future direction for the large-scale experiment.

## Methods

### Ethical matters

This study proposed a pilot study on the effect of olfactory stimulus with white musk, as white musk aromatic oil and evaluated the effects of an original blend fragrance using artificial musk developed by Next Day Co., Ltd. and Kyushu University (hereinafter referred to as “white musk”) toward the psychological and physiological responses of humans. This study was conducted with the approval of the Ethics Committee of the Faculty of Agriculture, Kyushu University (application number: 23 − 004, approval number: 110, project title: Exploratory Research on the Functionality of Fragrances, Approval Date: July 28, 2023. We also registered this study UMIN Clinical Trials Registry with register ID : UMIN000051972, August 24th, 2023 (24/08/2023). All measurements involving human participants were conducted in accordance with the Declaration of Helsinki. Prior to participation, participants were fully informed of the purpose and content of this study. After confirming that the participants fully understood and agreed with the content, we obtained their free and written informed consent to participate in this study.

### Participants

The total participants were 10 people, 5 man and 5 women (23.2 ± 2.3 years). The study design was a randomized, single-blind, crossover study. Participants were healthy adults without physical or mental problems. Participants were randomly assigned to two groups of 5 people and participated in the study twice. In Group A, the unscented was presented on the first test, and white musk was presented on the other day. In Group B, white musk was presented on the first test and the unscented on the other day. The washout period for the previous and subsequent tests was about 2 weeks. During the washout period, one participant was found to have the effects of COVID-19, and this participant is excluded from the physiological analysis in this study. The study was conducted from August 28 to October 4, 2023.

### Olfactory function confirmation

Prior to the experiment, an interview session was held with the participants to explain the experiment and to test olfactory function of the participant with three different aromas: lavender, white musk, and fir. During this meeting, participants reported that they were able to distinguish between the aromas. In addition, the aromas presented were preferred by all participants. During this session, we did not inform the participant what kind of aroma will be presented during the experiment to avoid the bias.

### Experimental room and olfactory simulation presentation

**Fig. 5:**
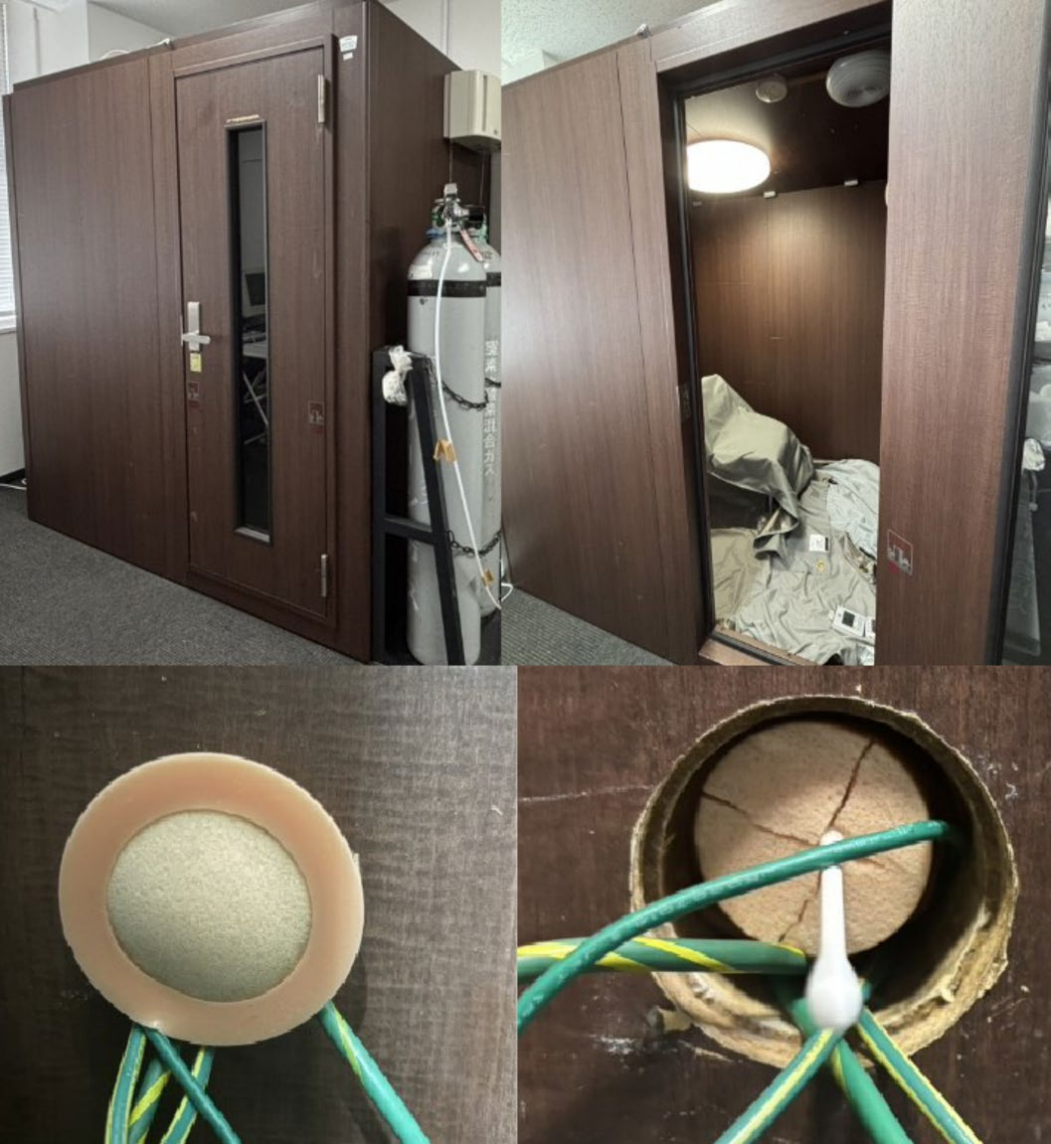
Experimental room and olfactory stimulus method.

In the human test, a soundproof room (width 1,700 mm, depth 1,260 mm, height 1,965 mm, manufactured by Yamaha Corporation) (hereinafter referred to as the laboratory) installed in Room 457 of West Building 5 on the Ito Campus of Kyushu University was used (Fig. 5). The test was conducted in a 4.2 m3 experimental space under conditions that suppressed external sound and light stimulation. The temperature and humidity before the start of the test were 22.4 ± 1.4℃ and 63.1 ± 5.5% (TR-72wf T&D Temperature and Humidity Data Logger, T&D Co., Ltd.), and the carbon dioxide concentration was 567.6 ± 118.8 ppm (testo 160 IAQ - Cloud monitoring logger). The white musk (undiluted solution) used in this study’s intervention is widely used in household detergents, perfumes, and cosmetics, and in product manufacturing, the undiluted solution is added to sprays at 1% and diffusers at 2–3%. To detect the scent, a cotton swab was soaked with 10 µL of white musk and inserted into the room through a hole made in the laboratory (Fig. 5). No fans were used during the test, and doors were not opened or closed to eliminate the effects of external sound and light. We divided the test condition into unscented and white musk aromatic oil inhalation. In addition, our participants were blinded to the type of scent presented, they did not know what scents were in the air in the room.

### Experimental procedure

**Fig. 6:**
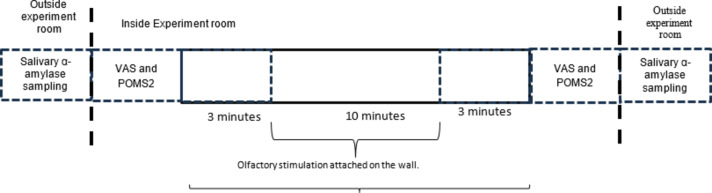
Experiment timeline.

Participants changed into a designated uniform before the start of the experiment to ensure uniform scent conditions. After being fitted with bioelectrodes, participants entered the experimental room and were seated in a chair. Two types of questionnaires were administered to all participants at pre- and post-measurement: a visual analog scale (VAS) of mood and a short version of the Profile of Mood States Second Edition (POMS2) Japanese version. An examination was performed. During the electroencephalogram and electrocardiogram measurements in the laboratory, participants maintained a resting state with their eyes closed for a total of 16 min. In the white musk aroma condition, the conditions were divided into 3 conditions, they are: 3 min before the olfactory stimulus, 10 min during the olfactory stimulus, and 3 min after the olfactory stimulus was removed from the room wall. Electrical activity was recorded. Salivary amylase measurements were also taken before the electrodes were applied to the participants and after the electrodes were removed as shown in Fig. 6. Room temperature before the start of the experiment was monitored and the result showed the average condition as follows: 22.4 ± 1.4℃, humidity: 63.1 ± 5.5%.

### Chemical profiling analysis in the white musk aromatic oil

A gas chromatography mass spectrometry system (GC-MS; 7890 A/5975 C, Agilent Technologies, Santa Clara, CA) with a thermal desorption unit (TDU; Gerstel, Mülheiman der Ruhr, Germany) was used for analysis of the white musk aromatic oil. TDU-GC-MS analysis condition is shown in Table 4. Diluted sample solution was injected in the splitless mode. GC-MS was equipped with a DB-5MS column (60 m×0.25 mm; 0.25 μm film thickness; Agilent Technologies). The oven temperature program was held at 70 °C for 1 min, gradually increased (40 °C/min) to 150 °C and held for 3 min, and then to 200 °C (5 °C/min) and increased (20 °C/min) to 300 °C and held for 3 min. Helium was used as the carrier gas at a flow rate of 1.2 mL/min. The synthetic musk compounds were identified by comparing the mass spectrum with the National Institute of Standards and Technology mass spectral library (NIST 11.0).

**Fig. 7:**
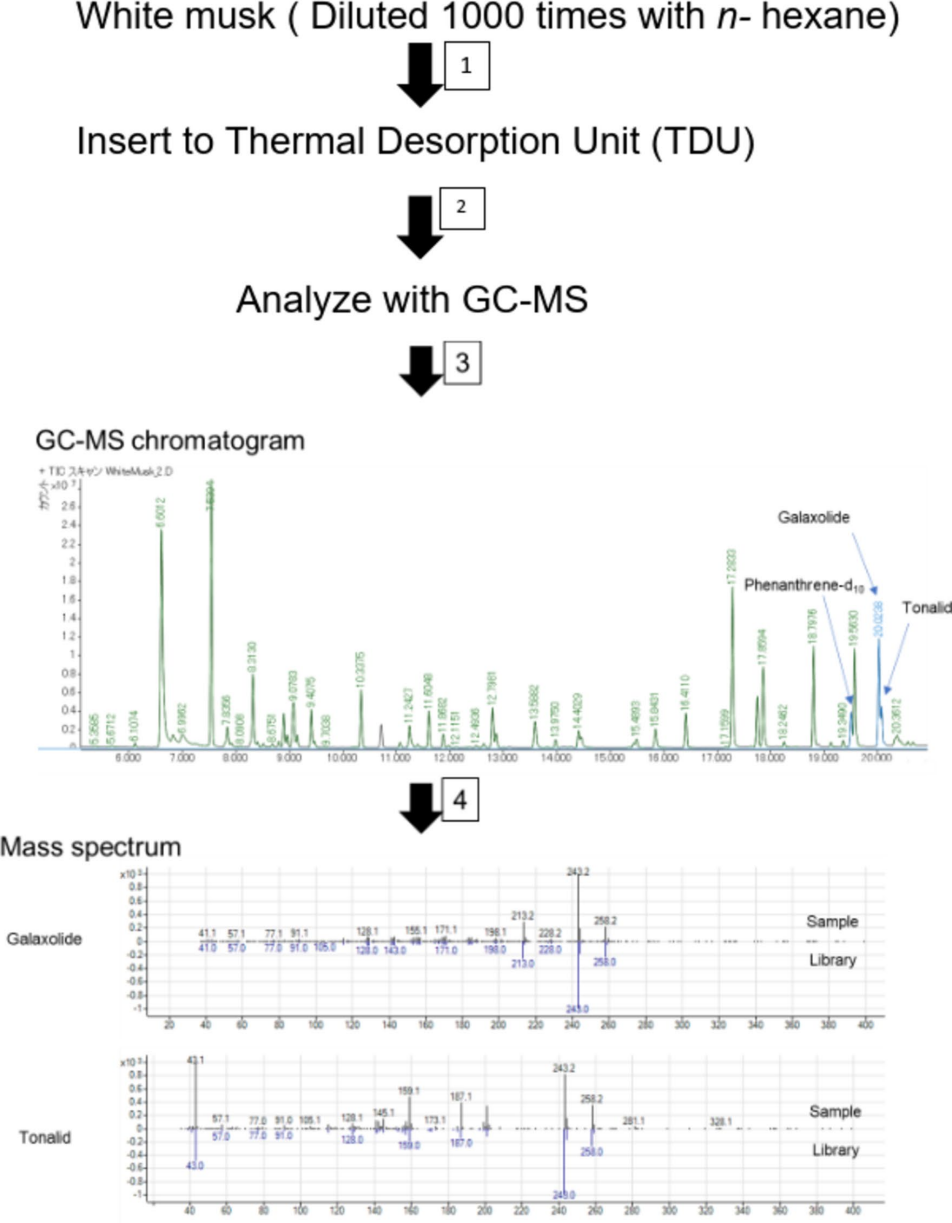
Chemical profiling analysis process of the white musk aromatic oil.

For quantitative analysis, the calibration curve was created by using standard substances and internal standard substance (IS). The standard substance galaxolide was purchased from BLD Pharmatech Ltd. (Shanghai, China), tonalide from Fluorochem Ltd. (Hadfield, UK), and IS phenanthrene-d_10_ from FUJIFILM Wako Pure Chemical Corporation (Osaka, Japan). One µL of the mixture of the standard substances and IS prepared to the predetermined concentration was added to the glass wool at the tip of the glass liner for TDU (length 60 mm, outer diameter 6 mm, inner diameter 5 mm, Gerstel, Mülheiman der Ruhr, Germany) with a micro-syringe and analyzed under the conditions shown in Table 5. One µL of the white musk solution diluted to 1000 times in hexane (FUJIFILM Wako Pure Chemical Corporation, Osaka, Japan) with IS was analyzed under the same conditions as above. The concentrations of galaxolide and tonalide were calculated using the internal standard method. The process of chemical profiling analysis was shown in Fig. 7. As shown in the GC-MS chromatogram in Fig. 7, several peaks were detected in the white musk aromatic oil that were combination from various synthetic fragrances. In this study, galaxolide and tonalide, which are the characteristic components of white musk, were selected for quantitative analysis.

**Table 5:** TDU-GC-MS analysis condition.

TDU	40 ℃ (0.1 min) - 720 ℃/ min ‐ Final temperature 220 ℃ (3 min) Transfer line to GC 220 ℃
CIS	100 ℃ (0.05 min) - 12 ℃/ s ‐ Final temperature 220 ℃ (10 min)
Carrier gas	He, Flow 1.2 mL/ min
Injection	Splitless mode
Column	DB-5MS: (Agilent Technologies, 60 m×0.25 mm i.d., 0.25 μm)
Oven	70 ℃ (1 min) - 40 ℃/ min − 150 ℃ (3 min) − 5 ℃/ min − 200 ℃ (0 min) − 20 ℃/ min − 300 ℃ (3 min)
Transfer line temperature	280 ℃
Ion source	230 ℃
Ionization	Electron impact 70 eV

### Psychological evaluation

An overview of the two questionnaire tests conducted is shown below.

#### Impression survey toward presented aroma

VAS (Visual analogue scale) was used to investigate the impression of the scent in the room. VAS is a widely used assessment method for estimating the degree of pain and determining the degree of pain relief. Healthcare professionals ask patients to measure the degree of pain on a line drawn between two ends. It is a continuous measure consisting of horizontal or vertical lines, usually 100 mm long. In recent years, it has been widely used in the medical field to measure health-related quality of life. It has also been used to measure post-sleep alertness, quality of life, anxiety, breathlessness, nausea, dyspnea, pruritus intensity, and attitude toward the environment. In addition, its usefulness has been reported in numerous applications in research on education, sound environments, and scents.

In this test, the left end of a 10 cm straight line was ``no impression’’ (0), and the right end was ``impression’’ (100). Participants placed a mark on the line for each item as a breaking point. Table 6 shows the adjectives used as evaluation words in this test. The evaluation words were adjectives and verbs expressing fragrance impressions, and responses to a total of 21 items were estimated as a VAS score (%).

**Table 6:** VAS spatial scent impression questionnaire.

1. Like	8. Roasty	15. Elegant
2. Bitter	9. Fresh	16. Modern
3. Sweet	10. Relaxing	17. Smelly
4. Weak	11. Clear	18. Nostalgic
5. Astringent	12. Comfortable	19. Mild
6. Sour	13. Breezy	20. Calming
7. Strong	14. Refreshing	21. Gorgeous

#### Mood states change survey towards presented aroma

The Profile of Mood States Second Edition (POMS2) Japanese version (hereinafter referred to as POMS2 abbreviated version) (Kaneko Shobo) was used to evaluate mood states and compare the states before and after fragrance presentation. This is a revised version of POMS, and POMS2 is a questionnaire that can quickly assess not only relatively long-lasting emotions, but also fluctuating and transient emotions. “Anger – Hostility (AH)”, “Confusion – Bewilderment (CB)”, “Depression – Depressed (DD)”, “Fatigue – Intertia (FI)”, “Tension – Anxiety (TA)”, “Vigor – Activity (VA)”, “ The mood state in a predetermined time frame is evaluated from the 7 scales of “friendship (F)” and the “Total Mood Disturbance (TMD) score,” which comprehensively represents negative mood states. In this study, we used the shortened version of POMS2 to reduce the burden on participants. Participants responded to a total of 35 questions on 7 scales, 5 items each, asking how they felt right now, ranging from ``Not at all’’ (0 points) to ``Very much’’ (4 points).) Answers were given on a five-point scale. The total score for each subscale was calculated, and the score for “Vibrance-Vitality” was subtracted from the total score for “Anger-Hostility,” “Confusion-Embarrassment,” “Depression-Depressed,” “Fatigue-Apathy,” and “Tension-Anxiety.” A “TMD score” was calculated. POMS2 short version scores were converted into standardized scores (T-scores) to assess participants’ mood states.

### Physiological evaluation

Physiological evaluation was performed by salivary amylase and by measuring electrocardiograms (ECG) and electroencephalograms (EEG). During the analysis of ECG and EEG, one participant has been excluded due to the recovery from illness. An overview of each is shown below.

### Measurement of salivary amylase activity

Salivary α-amylase was measured by salivary α-amylase monitor (Nipro Co.). Salivary α-amylase was used as an indicator of stress marker because it increases with the acceleration of sympathetic nerve activity.

### Measurement of autonomic system

ECG was measured by PolymateV (Miyuki Giken Co.) with sampling frequency 1000 Hz. High frequency components (HF) and low frequency components (LF) of electrocardiogram (ECG) were calculated by power spectral analysis of R-R intervals and used as an indicator of autonomic nerve activity. In this experiment, frequency analysis was performed from the RR interval data obtained by measuring electrocardiograms, HF and LF/HF were calculated, and the influence of fragrance on sympathetic and parasympathetic nerve activity was evaluated. The changes in RRI of the electrocardiogram every 10 s were calculated, and HF and LF/HF values were calculated using a CD method R-R interval analysis program (NoruPro Light Systems, R-R Interval Analysis).

### Brain activity measurement

**Fig. 8:**
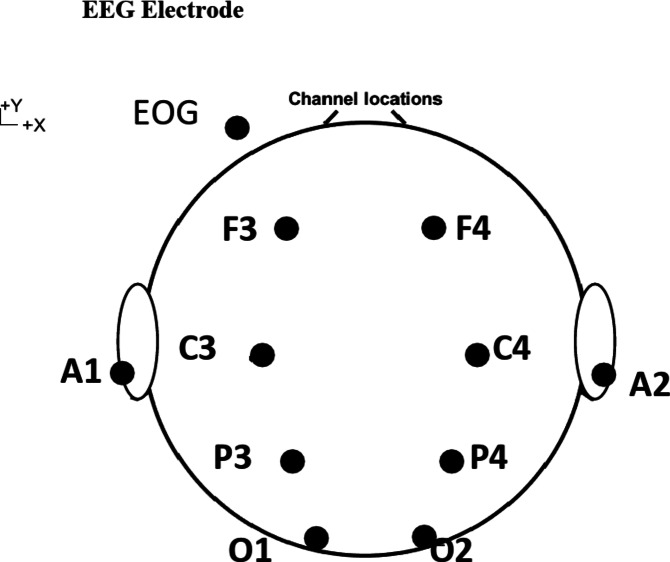
EEG electrode placement.

In this study, a biological amplifier (Polymate V, Miyuki Giken) was used to measure brain waves as in electrocardiogram measurements. The sampling rate was 1000 Hz. Based on the international 10-20 method, recording electrodes were placed at F3 (left side of the frontal region), F4 (right side of the frontal region), C3 (left side of the central region), and C4 to confirm the distribution of EEG on the scalp with a small number of electrodes. (right side of the center), P3 (left side of the parietal region), P4 (right side of the parietal region), O1 (left side of the occipital region), and O2 (right side of the occipital region) (Fig. 8). Reference electrodes were placed on A1 (left earlobe) and A2 (right earlobe), and A1 and A2 were connected. Biological signal active electrodes with an amplifier were used to record low-noise brain waves even at high impedance. The electrodes were fixed to the scalp with bioelectric signal measurement paste so that the resistance was 20 kΩ or less. In this experiment, participants maintained a resting state with their eyes closed during the EEG measurements. To observe whether blink noise was mixed into the brain waves, electrodes were placed on the upper, lower, and left sides of the left eye and on the right side of the right eye, and electrooculograms (EOGs) were measured. An electrooculogram measures the corneal-retinal potential of the eyeball (the corneal side is positively charged, and the retinal side is negatively charged), which changes as the eyeball moves. A vertical electrooculogram (VEOG) and a horizontal electrooculogram (HEOG) were recorded. During EEG recording, clothlike electromagnetic shields (Noi Cut Sheet 44552, GE Healthcare Japan, Tokyo) were placed around the participant’s feet, chair, and EEG monitor to suppress commercial power noise from the participant’s feet. Brain analysis was performed in the MATLAB 2013b^36^ environment using the EEGlab^37^ toolbox to plot brain topography (such as Fig. 3) and a homemade script to detect 1/f fluctuations, the process of detection as shown in Supplementary Fig. 1.

### Statistical analysis

Regarding between-group comparisons of before-after differences and changes in white musk presentation and unscented (no fragrance presentation) groups, normality was confirmed using the Shapiro-Wilk test, and paired t was used for comparison pairs where normality could be assumed. A two-tailed test was conducted. For comparison pairs for which normality could not be assumed by the Shapiro-Wilk test, the Wilcoxon signed-rank test was performed. The significance level was set at 5% using a two-tailed test.

## Electronic supplementary material

Below is the link to the electronic supplementary material.

Supplementary Material 1

## Data Availability

The data sets used and/or analyzed in this study are available from the first author or the corresponding author upon reasonable request.
