# Supplementary material for: A pilot study on the effects of olfactory stimulation with white musk aromatic oil on psychophysiological activity: a crossover study
Source: Sci Rep. 2025 Jan 11;15:1723. doi: 10.1038/s41598-024-83887-2 (PMC11724847; doi:10.1038/s41598-024-83887-2)
Supplement: Supplementary file 1 — Supplementary Material 1 [file 41598_2024_83887_MOESM1_ESM.docx]

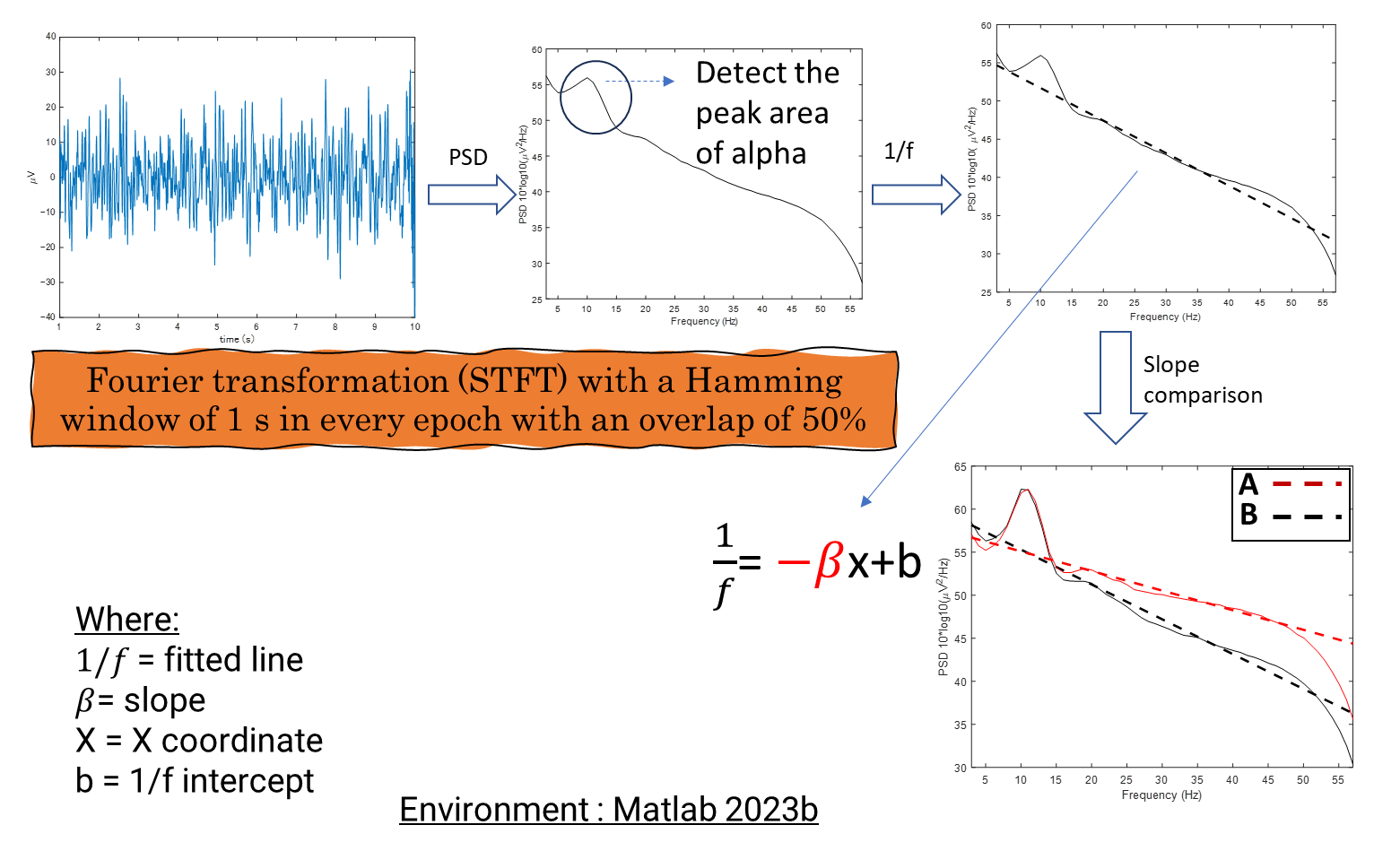


Supplementary Figure 1. The process of detecting 1/f fluctuations starting from the raw data, then converted to frequency domain by using Short Time Fourier Transform (STFT) with Hamming window of 1s in each epoch, with overlap of 50%. After obtaining the image of power spectral density, the highest peak of alpha band is detected, linear fitting line is calculated.

Supplementary Figure.1 The detection process of 1/f fluctuations
